# Comparison of the Burden of Falls Among Older Adults Aged 70 Years and Over Between Global and China

**DOI:** 10.1093/geroni/igaf122.3456

**Published:** 2025-12-31

**Authors:** Xiaodong Chen, Liping Li

**Affiliations:** Shantou University, Shantou, Guangdong, China (People’s Republic); Shantou University, Shantou, Guangdong, China (People’s Republic)

## Abstract

**Objective:**

To estimate the burden of falls in older adults globally and in China, and project the burden through 2030.

**Methods:**

Using data from the Global Burden of Disease Study 2021, we calculated age-standardized incidence rate (ASIR), mortality rate (ASMR), and disability-adjusted life years (ASDR) for falls in people aged ≥70 years from 1990 to 2021. Joinpoint regression was used to analyze the trend of falls disease burden during 1990-2021, and Bayesian age-period-cohort (BAPC) modeling was used to predict the trend of falls disease burden in global and China during 2022-2030.

**Results:**

From 1990 to 2021, the global burden of falls increased, with AAPCs of 0.60 for ASIR, 0.69 for ASMR, and 0.30 for ASDR. In China, the increase was more pronounced, with AAPCs of 2.44, 1.47, and 0.89, respectively. Projections for 2022-2030 show global ASIR will rise from 7,672.94 to 8,036.87 per 100,000, while ASMR will decrease from 106.72 to 96.96. In China, ASIR will increase from 7,143.75 to 8,757.76, and ASMR will decrease from 92.52 to 84.95. The global ASDR will decrease from 2,795.73 to 2,711.72, while China’s ASDR will rise from 2,293.30 to 2,503.68.

**Conclusions:**

The burden of falls disease continues to increase globally and in China in the older adult population aged 70 years and older, with a significant rise in ASIR in China in particular. Compared to global, more attention should be paid to falls ASIR and ASDR in China in the future.

